# Protective Effects of Bone Mineral Density on Age‐Related Macular Degeneration: A Mendelian Randomization Study

**DOI:** 10.1155/ije/6619808

**Published:** 2026-01-23

**Authors:** Yuxin Sun, Junfei Huang, Ziran Zhang, Zejun Chen, Zhengran Li, Fanye Wu, Zijin Wang, Tong Wu, Guoguo Yi, Fanke Meng, Shuduan Wu

**Affiliations:** ^1^ The Second Clinical Medicine School, Southern Medical University, Guangzhou, China, fimmu.com; ^2^ Ophthalmology Department, Zhujiang Hospital, Southern Medical University, Guangzhou, China, fimmu.com; ^3^ The First Clinical Medicine School, Southern Medical University, Guangzhou, China, fimmu.com; ^4^ Ophthalmology Department, The Sixth Affiliated Hospital, Sun Yat-Sen University, Guangzhou, China, sysu.edu.cn; ^5^ Biomedical Innovation Center, The Sixth Affiliated Hospital, Sun Yat-Sen University, Guangzhou, China, sysu.edu.cn; ^6^ Emergency Department, Zhujiang Hospital of Southern Medical University, Guangzhou, China, fimmu.com

**Keywords:** age-related macular degeneration, bone mineral density, Mendelian randomization, osteoporosis

## Abstract

**Introduction:**

Observational studies have established the connection between a decrease in bone mineral density (BMD) and an increased susceptibility to age‐related macular degeneration (AMD). However, the cause‐and‐effect link of this correlation remains uncertain. This study employed Mendelian randomization (MR) methods to examine the causality between BMDs and AMD.

**Materials and Methods:**

The GEnetic Factors for OSteoporosis (GEFOS) Consortium, UK Biobank, and FinnGen Biobank offered summary statistics for BMD and AMD. We adopted an array of quality control procedures to screen for eligible instrumental single nucleotide polymorphisms (SNPs). The inverse variance weighting (IVW) algorithm was the most trustworthy approach in MR analyses. Furthermore, we employed additional analytical methods such as MR‐Egger and weighted median (WM) for confirming the soundness of the present MR outcomes.

**Results:**

The findings indicated that genetically predicted total body BMD (TB‐BMD, IVW: OR = 0.772, 95% CI = 0.642–0.928, *p* = 0.006), lumbar spine BMD (LS‐BMD, IVW: OR = 0.739, 95% CI = 0.583–0.937, *p* = 0.013), femoral neck (FN‐BMD, WM: OR = 0.626, 95% CI = 0.432–0.906, *p* = 0.013), and TB‐BMD (age over 60, MR‐Egger, OR = 0.383, 95% CI = 0.163–0.902, *p* = 0.041) were associated with lower odds of wet AMD. No significant causal relationship can be found between other BMDs and Wet‐AMD, or between BMDs and Dry‐AMD.

**Conclusion:**

Our MR analysis supported the causal correlation between genetically predicted BMD and Wet‐AMD. As to Dry‐AMD, it was not causally related to BMD. Our study complemented the evidence from previous observational surveys and emphasized the extraordinary importance of monitoring BMD for preventing and treating AMD.

## 1. Introduction

Afflicting 196 million people globally [1], age‐related macular degeneration (AMD) is the primary contributor to irreversible eyesight deterioration in elderly individuals and is forecast to impact an estimated 288 million people internationally by the Year 2040 [2]. Crucially, the societal burden of AMD is increasing due to aging populations and increased longevity [3]. The key characteristic of AMD is the aggregation of sediments outside the cells (drusen), as well as the regression of photoreceptors and adjoining organizations [4]. Advanced AMD can be subdivided into Wet‐AMD (distinguished by the formation of new blood vessels in the choroid) and Dry‐AMD (called geographic atrophy) [5].

As the world’s fourth most prevalent reason for blindness, individuals suffering from serious AMD report a 63% decrease in life quality, and the disease’s treatment entails huge financial demands [6]. With limited options for the prevention and treatment of AMD, research on pathogenesis and pathophysiology is imperative to identify novel therapeutic targets for AMD. As of today, there are strong reasons to believe that natural aging changes drive AMD progression [7]. Nonetheless, the underlying mechanisms remain incompletely elucidated. Upon investigating the mechanisms, we observed a correlation between osteoporosis (OS) and an elevated susceptibility to AMD. OS, the most common age‐related metabolic skeletal disorder, is marked by reduced skeletal strength, deteriorated microstructure, and enhanced risk of fractures [8]. OS afflicts more than 200 million individuals throughout the world, placing one in five men and one in three women older than 50 at risk of fracturing their bones [9]. Presently, the diagnosis of OS involves the assessment of bone mineral density (BMD) using dual‐energy X‐ray absorptiometry (DXA) [8]. The World Health Organization (WHO) describes OS as BMD at least 2.5 standardized errors lower than the mean among young, physically fit women [10].

Both AMD and OS (or BMD loss) are characteristics closely associated with aging, and they share multiple triggers, including tobacco use, diabetes, and inadequate vitamin D levels [11]. Additionally, they are both chronic inflammatory diseases associated with inflammatory pathway genes [12]. With common links in pathogenic mechanisms, there has been consistent intrigue on the association between AMD and OS (or BMD loss). A multicenter cross‐sectional study in the United States found an established connection between high BMD and a decreased chance of AMD in older women [13]. A study utilizing the National Health and Nutrition Examination Survey discovered a correlation between the intake of more minerals to prevent OS and a higher occurrence of AMD [14]. In a large study of the Asian population, OS influences the progression of AMD within women, and there is a linear correlation between femoral neck OS and AMD in women [15]. However, a long‐term longitudinal follow‐up study has found that both men and women who have OS are more likely to get AMD [16]. The confirmation of a direct causal connection between BMD and AMD is still pending. The inconsistent results may be attributed to differences in populations, limited samples, and confounding factors. Therefore, exploring the projected genetic causality between BMD and AMD through a novel approach is urgently required.

Mendelian randomization (MR) serves for identifying the causality between exposures and outcomes using a variation of inheritance as instrumental variables (IVs), commonly single nucleotide polymorphisms (SNPs) [3]. MR follows Mendelian’s second law, where alleles separate independently and are transmitted to the offspring randomly at the time of gamete formation [17]. Because genetic variation is fixed at conception, MR overcomes some critical limitations present in conventional observational studies, including confounding factors and reverse causation [18]. Therefore, MR is particularly appropriate in exploring long‐term causality. Herein, we carried out MR for probing a causation between BMD and AMD (including Wet‐AMD and Dry‐AMD). BMD included the total body BMD (TB‐BMD) and the other four sites (heel, lumbar spine, forearm, and femoral neck). To account for the influence of age on TB‐BMD, we conducted separate analyses for different age groups (age 0–15, 15–30, 30–45, 45–60, and over 60). Considering that the National Osteoporosis Foundation (NOF) recommends utilizing the lumbar spine BMD (LS‐BMD) and the femoral neck BMD (FN‐BMD) to define OS, and due to the potential impact of sex, we made a sex‐specific analysis among LS‐BMD and FN‐BMD [19]. Current AMD guidelines include no recommendations for screening for OS or BMD. Therefore, it is of extraordinary significance to investigate the causality between BMD and AMD to prevent and treat AMD.

## 2. Materials and Methods

### 2.1. Research Framework

**Figure 1:**
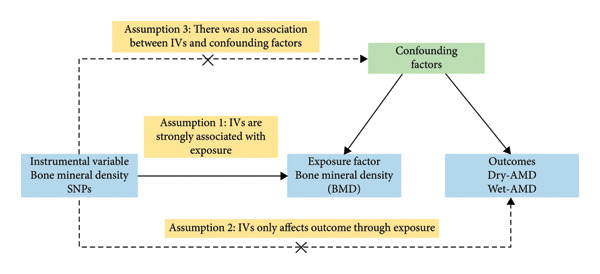
The requirements and framework of MR. Three essential assumptions are required in MR analysis. Assumption 1: IVs are strongly associated with exposure. Assumption 2: IVs only affect outcomes through exposure, not other pathways. Assumption 3: There was no association between IVs and confounding factors. Dry‐AMD, dry age‐related macular degeneration; Wet‐AMD, wet age‐related macular degeneration; MR, Mendelian randomization.

**Figure 2:**
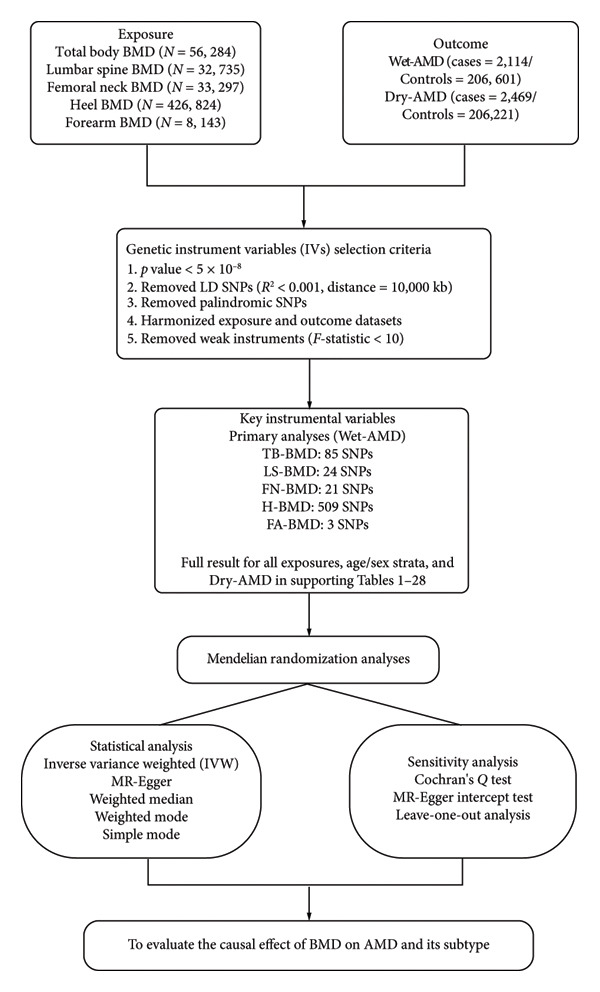
Flowchart of the Mendelian randomization analysis for the causal relationship between bone mineral density and age‐related macular degeneration. BMD, bone mineral density; AMD, age‐related macular degeneration; SNP, single nucleotide polymorphism; IVW, inverse variance weighted; LD, linkage disequilibrium; TB‐BMD, total body bone mineral density; LS‐BMD, lumbar spine bone mineral density; FN‐BMD, femoral neck bone mineral density; H‐BMD, heel bone mineral density; FA‐BMD, forearm bone mineral density; Wet‐AMD, wet age‐related macular degeneration; Dry‐AMD, dry age‐related macular degeneration.

The binary MR framework was utilized to evaluate whether BMD constitutes a potential hazard for Dry‐AMD and Wet‐AMD. Genetic variants as IVs came from SNPs in the genome‐wide association study (GWAS). The application of MR requires three essential conditions [20]: (1) variance in genes is highly linked to exposure (BMD); (2) variance in genes only affects outcome through exposure (AMD); (3) there is no connection established between genetic variation and any factors that could cause confusion or bias. This study followed STROBE‐MR as well as “Guidelines for performing MR investigations” [21, 22]. Figure 1 displays the MR framework utilized in this investigation. The specific data sources, analytical steps, and sensitivity analyses employed in this study are detailed in the flowchart presented in Figure 2.

### 2.2. Data Sources

The meta‐analysis provided summary data for TB‐BMD (*n* = 56,284), which contained adjustments for sexes, ages, BMI, genome primary components, and additional variables (such as evaluation center) [23]. The summarized statistics for LS‐BMD (*n* = 32,735), FN‐BMD (*n* = 33,297), and forearm BMD (FA‐BMD, *n* = 8143) were attained through the genetic factors for osteoporosis (GEFOS) (http://www.gefos.org/?q=content/data-release-2015;content/data-release-2015). After accounting for sexes, ages, age squared, and body mass, the initial study established standardized inherited metrics for 53,236 European participants [24]. The GWAS for heel BMD (H‐BMD) originated from the UK Biobank, comprising a total of 426,824 subjects, and was measured by quantitative ultrasounds. The original analysis included sex, age, genotyping array, and evaluation center as covariates in the fixed model [25].

The GEFOS website (http://www.gefos.org/?q=content/data-release-2012;content/data-release-2012) provided sex‐specific FN‐BMD and LS‐BMD databases. The findings were derived from a meta‐analysis that included 13 research types for FN‐BMD in men and 16 studies for FN‐BMD in women. Additionally, 12 studies were included for LS‐BMD in men and 13 studies for LS‐BMD in women [26]. Prior to the meta‐analysis, every person’s GWAS was adjusted for genomic controls [27].

Aggregated GWAS data for different age groups in TB‐BMD were offered by a meta‐analysis that included 30 GWASs and can be downloaded from GEFOS (http://www.gefos.org/?q=content/gefos-lifecourse-tb-bmd-gwas-results;content/gefos-lifecourse-tb-bmd-gwas-results) [23]. The 66,628 participants were divided into five age groups: age over 60 (*n* = 22,504), age 45–60 (*n* = 18,805), age 30–45 (*n* = 10,062), age 15–30 (*n* = 4180), and age 0–15 (*n* = 11,807). The TB‐BMD, LS‐BMD, FA‐BMD, and FN‐BMD were assessed using DXA, following established guidelines provided by the manufacturer. The International Society for Clinical Densitometry suggests using total body less head (TBLH) for assessment in pediatric cohorts (such as age 0–15) [23].

The GWAS databases for AMD can be downloaded through FinnGen Biobank GWAS (https://www.r5.finngen.fi/) and provide encapsulating comprehensive information on Wet‐AMD (2114 cases and 206,601 controls) and Dry‐AMD (2469 cases and 206,221 controls). More details on subject inclusion and exclusion criteria, research methods, and quality assurance are available in the initial study [23–26]. All information was gathered from previously released research. Therefore, no further ethical approvals or informed consent was required. All MR analyses were performed only on people of European descent, with the exception of the individuals who participated in the five age‐group TB‐BMD analyses. Table 1 presents the details of the databases used in our study.

**Table 1:** BMDs and AMD GWAS summarized databases.

Phenotype	Year	GWAS ID	Ancestor	Database	PMID
*Exposure*					
TB‐BMD	2018	ebi‐a‐GCST005348	86% European	GEFOS	29,304,378
H‐BMD	2019	ebi‐a‐GCST006979	European	UKB	30,598,549
LS‐BMD	2015	ieu‐a‐982	European	GEFOS	26,367,794
FA‐BMD	2015	ieu‐a‐977	European	GEFOS	26,367,794
FN‐BMD	2015	ieu‐a‐980	European	GEFOS	26,367,794
TB‐BMD (age 0–15)	2018	ebi‐a‐GCST005345	86% European	GEFOS	29,304,378
TB‐BMD (age15–30)	2018	ebi‐a‐GCST005344	86% European	GEFOS	29,304,378
TB‐BMD (age 30–45)	2018	ebi‐a‐GCST005346	86% European	GEFOS	29,304,378
TB‐BMD (age 45–60)	2018	ebi‐a‐GCST005350	86% European	GEFOS	29,304,378
TB‐BMD (age over 60)	2018	ebi‐a‐GCST005349	86% European	GEFOS	29,304,378
LS‐BMD (men)	2012	—	European	GEFOS	22,504,420
LS‐BMD (women)	2012	—	European	GEFOS	22,504,420
FN‐BMD (men)	2012	—	European	GEFOS	22,504,420
FN‐BMD (women)	2012	—	European	GEFOS	22,504,420

*Outcome*					
Dry‐AMD	2021	finn‐b‐DRY_AMD	European	FinnGen	—
Wet‐AMD	2021	finn‐b‐WET_AMD	European	FinnGen	—

*Note:* GWAS ID, genome‐wide association study identity; PMID: PubMed identity; H‐BMD, bone mineral density of heel; LS‐BMD, bone mineral density of lumbar spine; FA‐BMD, bone mineral density of forearm; FN‐BMD, bone mineral density of femoral neck; Dry‐AMD, dry age‐related macular degeneration; Wet‐AMD, wet age‐related macular degeneration; GEFOS, the genetic factors for osteoporosis; FinnGen, FinnGen Biobank.

Abbreviation: TB‐BMD, total body bone mineral density.

### 2.3. Selection of SNPs

The IVs for this study were SNPs obtained from GWASs. The selection of IVs should be based on the following principles [17]. Firstly, we should ensure that SNPs are tightly connected to exposure (BMD) and confirm their relevance over the entire genome with a threshold of *p* < 5 × 10^−8^. Secondly, we only chose independent SNPs with no linkage disequilibrium (LD), as determined by an *R* ^2^ value of 0.001 and a distance of 10,000 kilobases (kb). Thirdly, we performed the calculation of the *F* statistics to assess how closely SNPs are related to exposure (BMD) by the formula (*R* ^2^/(1 − *R* ^2^)) × ((*N* − *K* − 1)/*K*). The *R* ^2^ denotes the variability related to exposure (BMD) as interpreted using the selected SNPs, N represents the number of samples for the exposure (BMD), and *K* reflects the count of SNPs contained in each MR analysis. *F* statistics of certain SNPs below a threshold of 10 indicate a weak SNP and should be excluded.

Consequently, the number of instrumental SNPs retained for each MR analysis varied substantially. The largest number of SNPs (509) was utilized for the analyses of H‐BMD on both wet and dry AMD. In contrast, the smallest number of instruments was utilized for the analyses of TB‐BMD in the 15–30 age subgroups, with the model comprising a single SNP (rs10016972). The F statistics for all final IVs substantially exceeded the conventional threshold of 10, ranging from 19.6 to 8030.3. For the analyses relying on a single SNP, the *F* statistic was 32.1, well above the threshold, confirming its strength as an instrument. This effectively eliminates concern regarding weak instrument bias. Detailed information on selected SNPs and their association with outcomes (Wet‐AMD and Dry‐AMD) were provided in the Supporting Tables 1–28.

### 2.4. MR Analysis

The present research employed multiple methods that involved summarizing statistics to assess the causation between BMD and AMD, including different types of BMD measurements (TB‐BMD, H‐BMD, LS‐BMD, FA‐BMD, and FN‐BMD), as well as different subtypes of AMD (Dry‐AMD and Wet‐AMD). Among them, TB‐BMD was further classified into five age categories (age 0–15, 15–30, 30–45, age 45–60, and age over 60). LS‐BMD and FN‐BMD were further categorized based on sex, specifically into men and women.

In reporting the results, we prioritized the inverse variance weighted (IVW) method as the primary source of inference, as it provides the most accurate effect estimate when all IV assumptions are met. The weighted median (WM) method served as a robust supplementary approach, which provides consistent estimates even if up to 50% of the weight comes from invalid instruments. A causal effect was considered reliable and trustworthy when these primary methods yielded consistent and significant results, and when sensitivity analyses (e.g., MR‐Egger) did not detect significant horizontal pleiotropy.

IVW was the most trustworthy method if there was no directional pleiotropy. If there was heterogeneity, a random‐effects IVW model was utilized. Otherwise, a fixed‐effects model was applied. MR‐Egger is an adaptation of Egger regression; the intercept is often utilized to assess directional pleiotropy, indicated by a *p* value of less than 0.05. The WM was a supplementary method to give protection against ineffective IVs and produce dependable causal predictions, provided that at least 50% of all selected IVs proved to be genuine. Furthermore, we supplemented the analysis by incorporating both the simple mode and weighted mode.

### 2.5. Sensitivity Analysis

Heterogeneity and multidirectionality tests were conducted to secure the reliability and consistency of the findings. Firstly, MR‐Egger and IVW were used for heterogeneity analysis, and Cochran *Q* statistics were computed for the degree of heterogeneity (A *p* value below 0.05 indicated significant heterogeneity). Secondly, the MR‐Egger and intercept value evaluated the existence of lateral pleiotropy (*p* < 0.05 reflected significance). Pleiotropy, the phenomenon that a single IV can have an effect on several characteristics, is more likely when the intercept is away from zero. Additionally, an outlier SNP was identified using a “leave‐one‐out” test. When the results were significantly different after removing one SNP in turn for MR analysis, the SNP was considered an outlier.

All statistical analyses in this paper took place utilizing the “TwoSampleMR” package within R (Version 4.3.2, Vienna, Austria). Results were written as a 95% confidence interval (CI) and odds ratio (OR).

## 3. Results

### 3.1. Increases in TB‐BMD, LS‐BMD, and FN‐BMD Decreased the Odds of Having Wet‐AMD

**Figure 3:**
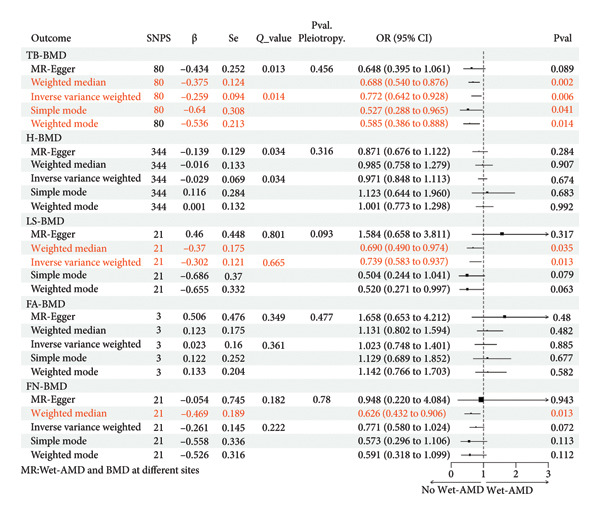
Causal associations between genetically predicted BMD (at different sites) on Wet‐AMD SNPs, single nucleotide polymorphisms; β, genetic effect sizes from exposure GWAS data; Se, standard error of effect sizes; *Q*_value, the *p* value of Cochran’s *Q* statistics test; OR, odds ratio; 95%. CI, 95% confidence interval; *P*val, *p* value; TB‐BMD, total body bone mineral density; H‐BMD, bone mineral density of heel; LS‐BMD, bone mineral density of lumbar spine; FA‐BMD, bone mineral density of forearm; FN‐BMD, bone mineral density of femoral neck; Wet‐AMD, wet age‐related macular degeneration.

The MR estimations for alternative approaches to assess the causality of BMD on Wet‐AMD are listed in Figure 3. The IVW analysis demonstrated a link of causality between innately determined TB‐BMD (OR = 0.772, 95% CI = 0.642–0.928, *p* = 0.006) and LS‐BMD (OR = 0.739, 95% CI = 0.583–0.937, *p* = 0.013) with Wet‐AMD. A rise in TB‐BMD by one standard deviation (SD) was associated with a 22.8% decreased odds of having Wet‐AMD, and a boost in LS‐BMD by one SD correlated with a 26.1% decreased odds of having Wet‐AMD. MR‐Egger showed no pleiotropy in these results (MR‐Egger: Pval. Pleiotropy > 0.05). The Cochran Q statistic suggested heterogeneity between TB‐BMD and Wet‐AMD (*p* = 0.013 < 0.05) in contrast to between LS‐BMD and Wet‐AMD (*p* = 0.801).

The WM is an additional technique that is often applied in the presence of heterogeneity. It yielded that increased TB‐BMD (OR = 0.688, 95% CI = 0.540–0.876, *p* = 0.002), LS‐BMD (OR = 0.690, 95% CI = 0.490–0.974, *p* = 0.035), and FN‐BMD (OR = 0.626, 95% CI = 0.432–0.906, *p* = 0.013) were positively linked to a reduced likelihood of developing Wet‐AMD. No significant causation can be observed between other BMDs and Wet‐AMD (H‐BMD: IVW, *p* = 0.674 > 0.05; FA‐BMD: IVW, *p* = 0.379 > 0.05). Therefore, we can conclude that TB‐BMD, LS‐BMD, and FN‐BMD have a definite causative impact on Wet‐AMD. Considering that the NOF recommends the use of LS‐BMD and FN‐BMD to define OS, and due to the potential influence of sex, we also performed a sex‐specific analysis. The results showed no significant causative impact of LS‐BMD and FN‐BMD on Wet‐AMD in men or in women (Supporting Tables 29–32).

### 3.2. An Increase in TB‐BMD (Age Over 60) Decreased the Odds of Having Wet‐AMD

**Figure 4:**
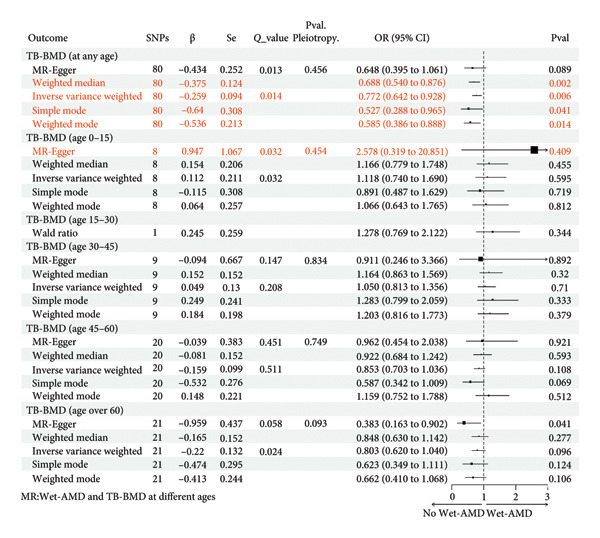
Causal associations between genetically predicted BMD (at different ages) on Wet‐AMD SNPs, single nucleotide polymorphisms; β, genetic effect sizes from exposure GWAS data; Se, standard error of effect sizes; *Q*_value, the *p* value of Cochran’s *Q* statistics test; OR, odds ratio; 95%. CI, 95% confidence interval; Pval, *p* value; TB‐BMD, total body bone mineral density; Wet‐AMD, wet age‐related macular degeneration.

We also analyzed the effect of age on the causality between TB‐BMD and Wet‐AMD. From the results shown in Figure 4, we found that an SD increase in TB‐BMD (age over 60) was related to a 61.7% decreased odds of having Wet‐AMD (MR‐Egger: OR = 0.383, 95% CI = 0.163–0.902, *p* = 0.041). While other approaches aligned with MR‐Egger’s, their outcomes did not yield statistically significant results (*p* > 0.05). In addition, there was no heterogeneity (*p* = 0.058 > 0.05) or pleiotropy (MR‐Egger: Pval. Pleiotropy = 0.093 > 0.05).

According to MR analysis, genetically predicted TB‐BMD (age 45–60) was associated with lower odds Wet‐AMD, while TB‐BMD (age 0–15, 15–30, and 30–45) was associated with higher odds Wet‐AMD. However, these associations were not statistically significant.

### 3.3. No Causality Between BMD and Dry‐AMD

**Figure 5:**
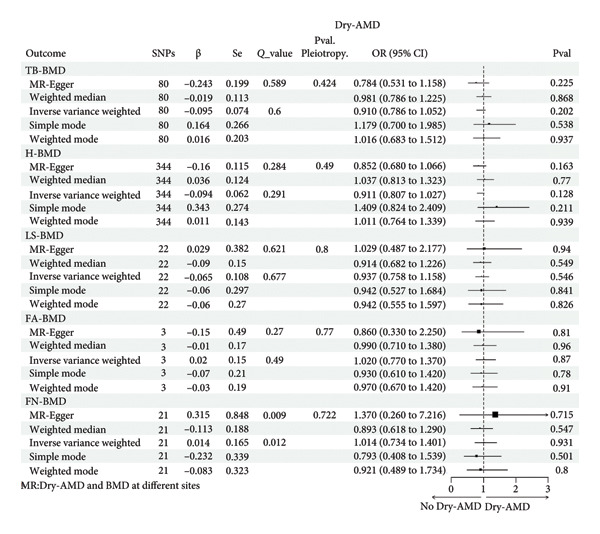
Causal associations between genetically predicted BMD (at different sites) on Dry‐AMD SNPs, single nucleotide polymorphisms; β, genetic effect sizes from exposure GWAS data; Se, standard error of effect sizes; *Q*_value, the *p* value of Cochran’s *Q* statistics test; OR, odds ratio; 95% CI, 95% confidence interval; Pval, *p* value; TB‐BMD, total body bone mineral density; H‐BMD, bone mineral density of heel; LS‐BMD, bone mineral density of lumbar spine; FA‐BMD, bone mineral density of forearm; FN‐BMD, bone mineral density of femoral neck; Wet‐AMD, wet age‐related macular degeneration.

Figure 5 displayed the findings of the analysis investigating the causative association between BMD at various locations and Dry‐AMD. We found no strong causal relationship between TB‐BMD (IVW, *p* = 0.202) and Dry‐AMD, as well as between H‐BMD (IVW, *p* = 0.128), LS‐BMD (IVW, *p* = 0.546), FA‐BMD (IVW, *p* = 0.27), and FN‐BMD (IVW, *p* = 0.931) and Dry‐AMD. The other 4 MR algorithms also showed no significant causal associations. The analysis of gender differences did not show a causal relationship either (Supporting Tables 33–36).

**Figure 6:**
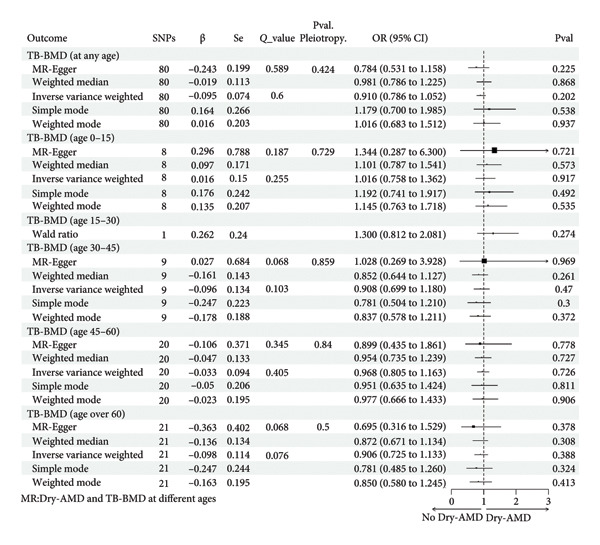
Causal associations between genetically predicted BMD (at different ages) on Dry‐AMD SNPs, single nucleotide polymorphisms; β, genetic effect sizes from exposure GWAS data; Se, standard error of effect sizes; *Q*_value, the *p* value of Cochran’s *Q* statistics test; OR, odds ratio; 95%. CI, 95% confidence interval; Pval, *p* value; TB‐BMD, total body bone mineral density; Dry‐AMD, dry‐age‐related macular degeneration.

Similarly, the analyses of the causality between TB‐ BMD at different ages and Dry‐AMD are shown in Figure 6. According to IVW or Wald ratio, TB‐BMD (age 0–15, IVW, *p* = 0.917; age 15–30, Wald ratio, *p* = 0.274; age 30–45, IVW, *p* = 0.470; age 45–60, IVW, *p* = 0.726; age over 60, IVW, *p* = 0.388) and Dry‐AMD were not considered to have a statistically meaningful causal connection.

### 3.4. Sensitivity Analysis

We calculated the *F* statistics and ensured that they were greater than 10 to verify the weak IV biases in the IVs chosen for BMD, Dry‐AMD, and Wet‐AMD. Heterogeneity and pleiotropy tests were applied to validate the robustness of the above MR analyses. The combination of the MR‐Egger and IVW was used for heterogeneity analysis, while the intercept value proved useful for the pleiotropy test. Although we found heterogeneity in some of the results, they were acceptable. According to the MR‐Egger intercept value, there was no pleiotropy in any of the above results (all *p* > 0.05). In addition, there was little change in the natural line after removing an SNP (all the natural lines were on the same side of 0). Therefore, leave‐one‐out sensitivity analyses suggested that our results were robust.

## 4. Discussion

Our present finding that BMD is causally associated with Wet‐AMD is novel. To the best of our knowledge, no MR study on the causal effect of BMD on AMD has yet been reported. The results suggested that genetically predicted increases in TB‐BMD, LS‐BMD, and FN‐BMD decreased the odds of having Wet‐AMD. Nevertheless, the impact of H‐BMD and FA‐BMD on Wet‐AMD did not show any significant causative effects. In addition, no significant causality between BMDs and Dry‐AMD was found.

### 4.1. Explanations for the Causality Between BMD (or OS) and AMD

BMD (or OS) was not significantly associated with other ocular diseases linked to aging (such as cataract, diabetic retinopathy, and open‐angle glaucoma) [15]. This phenomenon suggested that the specific effect of BMD on Wet‐AMD was not solely through the aging process (such as oxidative stress and cellular senescence). Several possible explanations for the causal relationship between BMD (OS) and Wet‐AMD are as follows.

Systemic inflammation in OS patients may raise the likelihood of developing Wet‐AMD. Patients with OS (or BMD loss) are often accompanied by a disruption in the balance of bone remodeling. This imbalance results in heightened osteoclast activity, reduced osteoblast activity, and elevated levels of cytokines that promote inflammation (such as IL‐1 and TNF‐α) in the circulation. The manipulation of these cytokines also profoundly impacts AMD [9]. Under the stimulation of proinflammatory cytokines, retinal pigment epithelium (RPE) cells release more cytokines (including IL‐1 and VEGF), which eventually leads to the occurrence of AMD [28]. Wet‐AMD is distinguished by the development of abnormal blood vessels in the choroid, mostly initiated and caused by VEGF [4]. Therefore, anti‐VEGF medication is predominantly employed to treat Wet‐AMD instead of Dry‐AMD. It is speculated that the causal effects of BMD on Wet‐AMD but not on Dry‐AMD (Figures 3 and 5) may be attributed to different roles of inflammation in the two types of AMD.

Bone morphogenetic protein‐4 (BMP‐4) is believed to play a significant role as a signaling molecule in skeletal development [29]. Furthermore, BMP‐4 is indispensable in eye morphogenesis and RPE specification [30, 31]. A study indicated that BMP‐4 may function as a molecular switch that determines whether patients with advanced AMD present with Dry‐AMD or Wet‐AMD [32]. Negatively regulated by TNF, the expression of BMP‐4 in RPE cells is reduced, and the deficiency of BMP‐4 signaling facilitates the angiogenic environment of Wet‐AMD [32]. However, BMP‐4 signaling leads to an upregulation of p53 and p21Cip1/WAF1 and a downregulation of pRb through Smad and p38 MAPK, thereby inducing lipofuscin accumulation and geographic loss in RPE with Dry‐AMD [33]. Therefore, the deficiency of BMP‐4 in patients with OS (or BMD loss) may contribute to the increased susceptibility to Wet‐AMD. To further validate this mechanistic link, our future work will include functional experiments in RPE cells to delineate the precise role of BMP‐4 signaling, alongside longitudinal MR analyses to assess the temporal dynamics of the causal relationship between BMD and AMD.

Furthermore, an independent MR study provides direct genetic support for our findings. That study confirmed a protective causal association between genetically predicted higher serum calcium concentrations and a lower risk of AMD [34]. Given that calcium homeostasis is central to maintaining BMD, this finding forms a logically consistent line of evidence with our result that genetically predicted higher BMD is protective against wet AMD. It suggests that genetic factors influencing calcium metabolism or skeletal homeostasis may collectively impact AMD risk through shared biological pathways, such as the regulation of angiogenesis or inflammation.

Hydroxylapatite [Ca5(PO4)3OH], which is ordinarily present in bones and teeth, has also been observed to build up in Bruch’s membrane in eyes affected by AMD [35]. OS (or BMD loss) may exert a stimulatory effect on the deposition of calcium and phosphate and the formation of drusen, which are composed of lipids, proteins, and minerals, leading to severe vision loss related to AMD [4, 36]. Additionally, vitamin D deficiency commonly accompanied by OS (or BMD loss) is suggested to accelerate mineral deposition in Bruch’s membrane (or sub‐RPE deposition) within AMD [15, 16]. It is reported that vitamin D is also a potent inhibitor of angiogenesis and oxidation, preventing the progression from early to Wet‐AMD [37]. The above studies indicate that decreased exposure to vitamin D and increased formation of drusen can account for the causality between OS (or BMD loss) and AMD.

### 4.2. New Possibilities for Treating AMD

Bisphosphonates (BPs), the most widely used treatment for OS, are available to increase BMD and decrease fracture risk, either by promoting apoptosis of osteoclasts or inhibiting bone resorption mediated by osteoclasts [38]. Interestingly, BPs inhibit choroidal neovascularization (CNV) in vivo and reduce VEGF expression of ARPE‐19 cells in vitro [39]. Apart from the negative effect on angiogenesis, BPs also increase the dissolution of hydroxyapatite crystals [40]. These findings propose novel possibilities for treating Wet‐AMD. Current therapies for Wet‐AMD predominantly involve surgical procedures such as lasers and intravitreal administration of drugs or antibodies [4]. Oral medications and eye drops are more acceptable to patients. However, several previous studies on the use of oral BPs for Wet‐AMD have yielded inconsistent results [40–42]. Although BPs have been reported to contribute to undesirable reactions such as uveitis and scleritis [43], their potential therapeutic effect on Wet‐AMD should not be ignored. Additional studies are necessary to mitigate adverse reactions and enhance the efficacy of BPs for treating retinal disorders.

### 4.3. Explanations for Variation in the Impact of BMDs on AMD

In our MR analysis, we also found that increases in LS‐BMD and FN‐BMD decreased the odds of having Wet‐AMD, while the effect of H‐BMD and FA‐BMD on Wet‐AMD was not significant. The adult skeleton has a ratio of 4:1 between cortical bone and trabecular bone [44]. The femoral neck consists mainly of cortical bone, while the lumbar spine comprises primarily of trabecular bone. The composition of the femoral neck is more closely proportional to that of the whole body, which may explain why it is more likely to be associated with AMD compared to the lumbar spine [45]. Another explanation is that the majority of BMD loss after the age of 65 years occurs primarily in the cortical bone rather than in the trabecular bone [46]. A study revealed that OS affecting the femoral neck had much more significant association with AMD than OS in the lumbar spine, which is consistent with our findings [15]. As a result, it has been inferred that differences in bone composition between skeletal sites may contribute to the variation in the impact of BMDs on AMD.

### 4.4. Advantages and Limitations

This study possesses distinct advantages. Primarily, it is the first to use genetic variants to detect causal associations between BMD (in different sites, sexes, and age groups) and AMD, to exclude the interference of confounders and reverse causation, and to compensate for the inherent limitations of observational studies. It is important to emphasize that we are conducting a causality analysis, not just a correlation analysis. Secondly, we subdivided Wet‐AMD and Dry‐AMD in our analysis due to their distinct pathological mechanisms. Based on current knowledge, although previous limited studies have found that OS (or BMD) was related to AMD, they have not resolved the critical debate on causality, nor have they examined the impact of OS upon the differential presentation of advanced AMD [15, 16]. Thirdly, we used a relatively large sample and multiple analyses to ensure the soundness of our findings. Additionally, it is advisable that physicians caring for OS patients should be aware of their heightened susceptibility to AMD and regularly evaluate their eyesight to guarantee early detection.

Nevertheless, this study still has some limitations. Firstly, the genetic instruments were derived from European‐ancestry GWAS summary data, and thus, the findings may not be directly generalizable to other populations. Known differences in LD patterns, SNP effect sizes, and allele frequencies across populations could affect the transferability of results. Future studies are needed to validate these findings in diverse ancestries. Secondly, the female gender also has an effect on the susceptibility to AMD in OS patients due to the greater vulnerability for acquiring AMD in postmenopausal women with OS [15]. However, we did not observe this specificity in women, which may be related to database selection, sample size, and ethnicity. More research is warranted in the future to explore this cause‐and‐effect connection. Thirdly, the biological functions of the selected SNPs and the associated diseases or behaviors are not fully elucidated in the present study. Our results only suggest a potential genetic causality, so it is critical to focus on the underlying mechanisms in the future to confirm the biological rationale.

Additionally, while observational studies suggest a link between postmenopausal OS and AMD, our sex‐stratified MR analysis found no significant association. Potential reasons include the following: (1) limited statistical power after sample size reduction due to stratification; (2) the fact that not all GWAS data for IVs were sex‐stratified, weakening the analysis; and (3) confounding by factors like hormone replacement therapy in observational studies, which our MR design might not capture.

In summary, our MR analysis suggests that genetically predicted increases in TB‐BMD, LS‐BMD, and FN‐BMD decrease the odds of having Wet‐AMD. It provides no evidence for causality between BMDs and Dry‐AMD. OS (or BMD loss) could induce or aggravate AMD through certain pathways. Thus, monitoring the BMD in patients with OS may be a good clinical practice to improve the prediction and prevention of AMD.

## Ethics Statement

All information was gathered from previously released research. Therefore, no further ethical approvals or informed consent was required.

## Conflicts of Interest

The authors declare no conflicts of interest.

## Author Contributions

Conceptualization: Yuxin Sun and Ziran Zhang; methodology: Tong Wu and Zijin Wang; formal analysis and investigation: Tong Wu and Zejun Chen; writing–original draft preparation: Yuxin Sun and Ziran Zhang; writing–review and editing: Fanye Wu, Zhengran Li, and Junfei Huang; resources: Shuduan Wu; supervision: Guoguo Yi, Fanke Meng, and Shuduan Wu. Yuxin Sun, Junfei Huang, Ziran Zhang contributed equally to the research.

## Funding

This study was supported by grants from the Guangdong Administration of Traditional Chinese Medicine (Project No. 20241201) and the Hospital Director’s General Research Fund (Project No. yzjj2023ms05).

## Supporting Information

Table 1. TB‐BMD exposure SNPs and their association with Wet‐AMD.

Table 2. H‐BMD exposure SNPs and their association with Wet‐AMD.

Table 3. LS‐BMD exposure SNPs and their association with Wet‐AMD.

Table 4. FN‐BMD exposure SNPs and their association with Wet‐AMD.

Table 5. FA‐BMD exposure SNPs and their association with Wet‐AMD.

Table 6. TB‐BMD (age 0–15) exposure SNPs and their association with Wet‐AMD.

Table 7. TB‐BMD (age15‐30) exposure SNPs and their association with Wet‐AMD.

Table 8. TB‐BMD (age30‐45) exposure SNPs and their association with Wet‐AMD.

Table 9. TB‐BMD (age 45–60) exposure SNPs and their association with Wet‐AMD.

Table 10. TB‐BMD (age over 60) exposure SNPs and their association with Wet‐AMD.

Table 11. LS‐BMD‐man exposure SNPs and their association with Wet‐AMD.

Table 12. LS‐BMD‐woman exposure SNPs and their association with Wet‐AMD.

Table 13. FN‐BMD‐man exposure SNPs and their association with Wet‐AMD.

Table 14. FN‐BMD‐woman exposure SNPs and their association with Wet‐AMD.

Table 15. TB‐BMD exposure SNPs and their association with Dry‐AMD.

Table 16. H‐BMD exposure SNPs and their association with Dry‐AMD.

Table 17. LS‐BMD exposure SNPs and their association with Dry‐AMD.

Table 18. FN‐BMD exposure SNPs and their association with Dry‐AMD.

Table 19. FA‐BMD exposure SNPs and their association with Dry‐AMD.

Table 20. TB‐BMD (age 0–15) exposure SNPs and their association with Dry‐AMD.

Table 21. TB‐BMD (age 15–30) exposure SNPs and their association with Dry‐AMD.

Table 22. TB‐BMD (age 30–45) exposure SNPs and their association with Dry‐AMD.

Table 23. TB‐BMD (age 45–60) exposure SNPs and their association with Dry‐AMD.

Table 24. TB‐BMD (age over 60) exposure SNPs and their association with Dry‐AMD.

Table 25. LS‐BMD‐man exposure SNPs and their association with Dry‐AMD.

Table 26. LS‐BMD‐woman exposure SNPs and their association with Dry‐AMD.

Table 27. FN‐BMD‐man exposure SNPs and their association with Dry‐AMD.

Table 28. FN‐BMD‐woman exposure SNPs and their association with Dry‐AMD.

Table 29. MR results of LS‐BMD‐man on Wet‐AMD.

Table 30. MR results of LS‐BMD‐woman on Wet‐AMD.

Table 31. MR results of FN‐BMD‐man on Wet‐AMD.

Table 32. MR results of FN‐BMD‐woman on Wet‐AMD.

Table 33. MR results of LS‐BMD‐man on Dry‐AMD.

Table 34. MR results of LS‐BMD‐woman on Dry‐AMD.

Table 35. MR results of FN‐BMD‐man on Dry‐AMD.

Table 36. MR results of FN‐BMD‐woman on Dry‐AMD.

## Supporting information


**Supporting Information** Additional supporting information can be found online in the Supporting Information section.

## Data Availability

The data that support the findings of this study are available in the supporting information of this article.
